# Riverine Landscape Patch Heterogeneity Drives Riparian Ant Assemblages in the Scioto River Basin, USA

**DOI:** 10.1371/journal.pone.0124807

**Published:** 2015-04-20

**Authors:** Paradzayi Tagwireyi, S. Mažeika P. Sullivan

**Affiliations:** School of Environment & Natural Resources, The Ohio State University, 2021 Coffey Rd., Columbus, OH, 43210, United States of America; INRA, FRANCE

## Abstract

Although the principles of landscape ecology are increasingly extended to include riverine landscapes, explicit applications are few. We investigated associations between patch heterogeneity and riparian ant assemblages at 12 riverine landscapes of the Scioto River, Ohio, USA, that represent urban/developed, agricultural, and mixed (primarily forested, but also wetland, grassland/fallow, and exurban) land-use settings. Using remotely-sensed and ground-collected data, we delineated riverine landscape patch types (crop, grass/herbaceous, gravel, lawn, mudflat, open water, shrub, swamp, and woody vegetation), computed patch metrics (area, density, edge, richness, and shape), and conducted coordinated sampling of surface-active Formicidae assemblages. Ant density and species richness was lower in agricultural riverine landscapes than at mixed or developed reaches (measured using *S* [total number of species], but not using Menhinick’s Index [*D*_M_]), whereas ant diversity (using the Berger-Park Index [*D_BP_*]) was highest in agricultural reaches. We found no differences in ant density, richness, or diversity among internal riverine landscape patches. However, certain characteristics of patches influenced ant communities. Patch shape and density were significant predictors of richness (*S*: *R* ^2^ = 0.72; *D*_M_: *R* ^2^=0.57). Patch area, edge, and shape emerged as important predictors of *D_BP_* (*R* ^2^ = 0.62) whereas patch area, edge, and density were strongly related to ant density (*R* ^2^ = 0.65). Non-metric multidimensional scaling and analysis of similarities distinguished ant assemblage composition in grass and swamp patches from crop, gravel, lawn, and shrub as well as ant assemblages in woody vegetation patches from crop, lawn, and gravel (stress = 0.18, *R* ^2^ = 0.64). These findings lend insight into the utility of landscape ecology to river science by providing evidence that spatial habitat patterns within riverine landscapes can influence assemblage characteristics of riparian arthropods.

## Introduction

Spurred in part by Wiens [1] guiding thesis that landscape ecology has important insights to offer aquatic ecology, the principles of landscape ecology have increasingly been applied to riverine systems [2–6]. In particular, the central role of patch dynamics (i.e., quality, connectivity, boundaries, context) can be aptly applied to riverine landscapes—conceptualized as the holistic ecological unit consisting of the main channel and slackwaters, the sub-bankfull inundation zone, and the suprabankfull inundation areas [7]—whereby the interaction of hydrology, sediment, and biotic factors form a rich mosaic of interconnected patches [8–10]. Riverine landscapes can exhibit a heterogeneous amalgam of patches including active and relict river channels, point bars, oxbow lakes, meander scrolls, natural levees, backwater sloughs, swamps, mud flats, and terraces, each representing a diversity of spatiotemporal dynamic successional stages. These spatiotemporal dynamics contribute to both lateral and longitudinal variation in biogeochemical processes, sedimentation, soil moisture, and elevation [11].

Hydrologic disturbance dynamics are of particular significance in riverine landscapes, where water movement represents a formative process linking aquatic and terrestrial “landscape” elements in both space and time [12,13]. For example, the dynamic flooding regime inherent to semiregulated or unregulated river-floodplain ecosystems is critical for patch heterogeneity [14,15]. Rising floodwaters connect the main channel to floodplain waterbodies (e.g., ponds, wetlands, slackwaters, etc.) and promote exchanges of aquatic biota, thereby exerting a homogenizing influence on aquatic communities [16,17]. Conversely, a mosaic structure is reestablished as floodplain waters recede, floodplain waterbodies are isolated, and aquatic communities become more heterogeneous. For terrestrial biota, flooding events reduce connectivity among patches and may increase within-patch heterogeneity as populations become isolated [18,19]. For high terrestrial biotic diversity to persist, a heterogeneous patch structure must remain after floodwaters have receded and connectivity has been reestablished.

In particular, terrestrial floodplain areas can be important habitats for riparian arthropods, including spiders, ground beetles, and ants [20–22]. Many riparian invertebrates have species-specific adaptations to disturbances associated with flooding, including timing of life-cycle stages and movement in and out of floodplain habitats [23]. Riparian invertebrate communities are often organized along both longitudinal and lateral gradients of soil moisture and elevation associated with floodplains [24]. For example, Paetzold, Yoshimura and Tockner [25] and Ballinger, Lake and Mac Nally [18] found that habitats that were affected by frequent flood inundation were almost devoid of arthropods immediately after flooding events. Thus, changes in flooding frequency and magnitude can cause variability in species abundance and assemblage composition [26]. As such, the complex interconnectivity of in-channel, riparian, and floodplain zones is thought to structure riparian arthropod communities [3,27].

In spite of significant conceptual advances in viewing river corridors as both internally heterogeneous and tightly linked to their surrounding landscapes, explicit applications of riverine landscape ecology are few [8] (but see, for example, Ballinger, Lake and Mac Nally [18] who used a landscape ecology approach to demonstrate that terrestrial invertebrates experience floodplains as landscape mosaics and Sullivan, Watzin and Keeton [28] who investigated habitat associations of riverine bird assemblages within a riverscape perspective). In this study, we investigated the associations between internal riverine landscape heterogeneity (i.e., patches) and the density, diversity, and composition of daytime, surface-active ant (Hymenoptera: Formicidae) assemblages within riverine landscapes in urban/developed, agricultural, and “mixed” (primarily forested, but also wetland and grassland/fallow, and exurban) areas of the Scioto River basin, Ohio, USA. At a coarse spatial resolution, we expected that developed and agricultural riverine landscapes, characterized by low hydrological connectivity between the floodplain and the main channel due to impoundments and or/channelization, would support low patch heterogeneity and low ant diversity. At a finer level of resolution, we hypothesized that specific patch types and characteristics within the riverine landscape (e.g., shape, size, connectivity, etc.) would influence ant assemblage density, diversity, and composition. For example, because of the documented associations between arthropod assemblages and floodplains (e.g., [18,20]), we anticipated that ant density and diversity would be higher in patches that experience reduced flood disturbances (e.g., woody vegetation patches) than in patches that experience more frequent and intense flood events (e.g., gravel bars, mudflats, swamps). The nested nature of our study design (i.e., patches and sites within landscapes) also allowed us test whether variation in riparian ant assemblages was driven by landscape-scale features or individual patch characteristics. This study represents an important proof of concept for the application of the principles of landscape ecology to riverine landscapes.

## Materials and Methods

### Ethics statement

Permission to access privately-owned land was given by all land owners. Field collections were carried out under a Wildlife Collection Permit issued by the Ohio Division of Wildlife (#15–49). Due to its focus on invertebrates, this study did not require any approval for animal care and use.

### Study area and site selection

At its confluence with the Ohio River, the Scioto River is a 6^th^-order, mixed-use system draining a 16,882-km^2^ catchment from its headwaters in central Ohio. The Scioto River catchment intersects three physiographic regions including the Till Plains, the Glaciated Appalachian Plateau, and the Unglaciated Allegheny Plateau [29,30]. Typical valleys of the Scioto River in our study area span ~2.5 km and form rich agricultural plains [31]. Channel gradient is typically low (~4 m/km), with pool-riffle morphology dominant in unmodified sections [31]. The Scioto River basin lies predominantly in a humid continental climate [32], receiving 900–1100 mm precipitation per year on average [33]. Land use and land cover (LULC) in the basin are dominated by cropland and pasture, which collectively comprise 59% of the catchment area [34]. However, the river also flows through multiple urban centers, including Columbus, Ohio with a population of 787,000 [35] as well as areas of mixed landscapes comprised of primarily deciduous forests but with minor percentages of small urban centers/towns, grassland, shrubland, forest, and wetlands [34]. The Olentangy River is the largest tributary of the Scioto River, joining the Scioto River in Columbus from the North.

**Fig 1:**
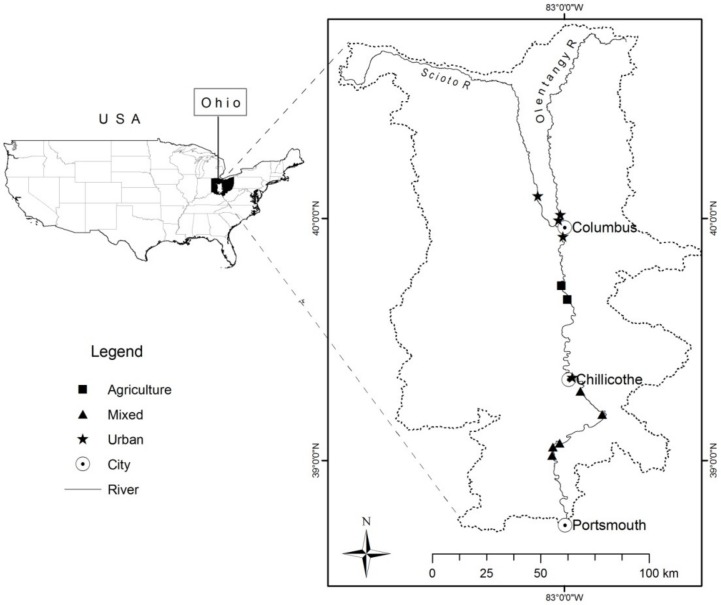
Location of the study system. The Scioto and Olentangy Rivers of the Scioto River basin of Ohio (USA) along with the twelve riverine landscape study reaches in agriculture, urban/developed, and mixed (forested, grassland, fallow, exurban) land-use classes.

Our study included 12 1,500-meter (m) riverine landscapes (i.e., study reaches) along ~200 km of the Scioto and Olentangy Rivers (Fig 1) that represented typical aquatic (e.g., flow, geomorphology) and riparian (e.g., vegetation, land use) characteristics of the river system at large. To select study reaches, we first used the National Land Cover Database [34] land-use maps in ArcGIS 10.1 (ESRI, Redlands, California, USA) to characterize LULC within a 500-m buffer of the main channel following Alberts, Sullivan and Kautza [36]. Subsequently, we classified study reaches as agricultural or developed if their adjacent riparian zones (within the 500-m buffer on each side and within the suprabankfull inundation area) were predominantly characterized by these LULC types (> 66% of total LULC by area, after Kawula [37]). Developed reaches were defined as riverine landscapes found in a city or town, with the most highly developed reaches located in and around Columbus, Ohio. Those riverine landscapes that had no predominant LULC were classified as “mixed”. Within each of these three land-use classifications (i.e., developed, agriculture, mixed), we then systematically selected five developed, five mixed, and two agricultural reaches. Although the study reaches were distributed along the length of the river, LULC patterns in the watershed and limited access to some stretches precluded a balanced design and equidistant sampling. Study reaches were separated by distance of 18.3 river km on average, although there was high variability (SD = 15.4 km).

### Riverine landscape patch metrics

We delineated riverine landscapes (defined here as the integrated ecological unit including the main channel, floodplain waterbodies, and the riparian zone) and characterized patches using a combination of remotely-sensed and field-collected data. Specifically, we used a combination of on-screen digitizing in ArcGIS 10.1 and Arc Pad 8.0 (Environmental Systems Research Institute: Redlands, California, USA) on a desktop computer and on a Personal Digital Assistant (Pharos 565 PDA, Pharos Science and Applications Inc., California, USA), respectively. The principal source of remotely-sensed data was the 2006, 30.48-cm resolution, natural color Digital Orthophoto Quarter Quadrangles of the study area obtained from the Ohio Statewide Imagery Program [38]. Using this approach and guidelines adapted from Holmes and Goebel [39] and Johansen, Phinn and Witte [40], we identified and digitized nine patch types in the riverine landscape (see Table 1). We then used Patch Analyst software [41] within a GIS to compute 17 patch metrics from which we selected 10 metrics that we deemed to be adequate representatives of overall patch patterns in order to characterize and quantify habitat patches at each of the riverine landscapes (Table 2).

**Table 1:** Riverine landscape patch types at the twelve Scioto and Olentangy River study reaches delineated from field and remotely-sensed data.

Patch Type	Description
Crop	Land tilled for crops including fallow areas.
Woody vegetation	Land covered by trees >6m in height.
Grass/Herbaceous	Grazed pasture.
Gravel	Bare/exposed soil, sand, or gravel along the main channel.
Lawn	Managed grass, particularly in recreational parks.
Mudflat	Exposed mud (wet soil) particularly along the main channel.
Open water	Surface water in main channel, floodplain waterbodies, and artificial impoundments (i.e., dams).
Shrub	Shrubs and young trees <6m in height.
Swamp	Herbaceous and woody marshes.

Patch classification was adapted from Johansen, Phinn and Witte [40].

**Table 2:** Patch metrics, measures, units, and descriptions used to quantify riverine landscape composition and configuration of the twelve study reaches of the Scioto and Olentangy Rivers, Ohio, USA.

Patch Metric	Measure	Unit	Description
**Area**	Total Land Area (TLA)	ha	Total area encompassed by riverine landscape.
	Mean Patch Size (MPS)	m^2^	Average size of all patches.
**Density**	Number of Patches (NP)	Num	Total number of patches.
**Edge**	Edge Density (ED)	m/ha	The length of all patch edges per riverine landscape area.
	Mean Patch Edge (MPE)	m	Average edge length of all patches.
	Total Edge (TE)	m	Total edge length of patches.
	Mean Perimeter Area Ratio (MPAR)	-	Mean of the ratio of each patch perimeter to its patch area.
**Richness**	Shannon Diversity Index (SDI)	-	Patch heterogeneity/diversity.
	Shannon Evenness Index (SEI)	-	Patch evenness (i.e., relative abundance and distribution of patch types).
**Shape**	Mean Shape Index (MSI)	-	Compares the patch shape to a square standard.

Detailed metric descriptions and formulas are provided in McGarigal and Marks [89].

### Ant surveys

**Fig 2:**
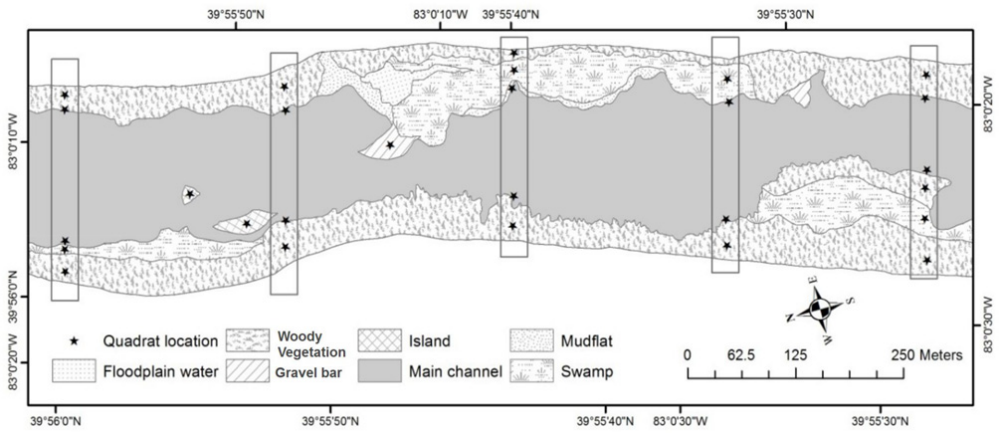
Experimental design. Example of experimental design at one of the study reaches including riverine landscape patches as well as transects and quadrats where ants were surveyed.

Terrestrial taxa that inhabit floodplain environments are often ubiquitous opportunists with general habitat requirements and the capacity to quickly recolonize after a disturbance event [24]. In particular, ants represent an excellent model taxon for this study because they respond rapidly to environmental change, represent a variety of trophic levels, are important ecosystem engineers and agents for plant seed dispersal, and have been used effectively as ecological indicators [42–45]. At each of our study riverine landscapes, we conducted surveys of surface-active ant assemblages between 11:00 and 16:00 once in June, once in July, and once in August of 2010–2012, such that each site was sampled for ants three times over the course of the study. We focused on surface-active ants as we presumed the species within this group would be most directly influenced by riverine patchiness as driven by hydrological disturbance (vs. arboreal species, for example). First, we established five longitudinal transects that were ~250 m apart and ran perpendicular to the main channel. Transects extended to the end of the riverine landscape, which we visually assessed primarily by breaks in slope and riparian-to-upland changes in vegetation and soils. Along each transect, we used a quadrat method (459 quadrats in total [46]) to survey ant assemblages at 3-m^2^ georeferenced plots distributed at the edge of the main channel and at locations within riverine landscape patches (Fig 2). Depending on transect length (which varied with width of riverine landscape, 311.7 m ± 89.2 m) and the number of distinct patches along each transect, we sampled from two to seven quadrats along each of five transects per reach. We also sampled additional quadrats at the centroids of distinct ecological patches (e.g., islands) that may have been missed by the systematic transect approach. All ants observed within or entering the quadrat in a 10-minute period [47] were counted and identified to species. Any ants that were difficult to identify in the field were collected, dispatched, and identified in the lab following Fisher and Cover [48] and AntWeb [49], consulting experts when necessary. Ant data from each sampling location were averaged across the three years for subsequent analysis.

### Numerical and statistical approach

For each quadrat, species richness *(S)* was estimated as the number of species sampled from the community. We also standardized *S* to Menhinick's Index (*D*_*M*_ *= S/√N*), which estimates species richness independent of sample size [50]. We estimated species diversity using the Berger-Parker Index [*(D*_*BP*_ *= N*_*max*_ */N*], where “*N*_*max*_” is the number of the most dominant species [51]. An increase in the value of *D*_*BP*_ accompanies a decrease in diversity and an increase in dominance. We also calculated ant density as the number of ants m^-2^. Because the raw patch metrics were at different scales of measurement, we standardized them to per unit variance (i.e., dividing each score by the standard deviation of each respective patch metric) and used the standardized scores in the statistical analysis [52].

First, we used analysis of variance (ANOVA) followed by Tukey-Kramer honestly significant difference (HDS) to test for differences in patch metrics among LULC types. We then examined the effects of LULC type (i.e., agricultural, developed, and mixed riverine landscapes) and patch metrics on ant assemblage density and diversity using nested ANOVA (patch type and site nested within LULC type, site included as a random factor). We also used non-metric multidimensional scaling (NMS) using Sorensen (Bray-Curtis) distance to analyze the partitioning of ant assemblage composition by patch type using a matrix of eight patch types (note that we excluded open water from all ant-patch analyses) and relative ant abundances. To compare differences in ant assemblage composition among patch types, we complemented NMS with analysis of similarities (ANOSIM) and Similarity Percentages (SIMPER), which were based on the Bray-Curtis index of dissimilarity, with 999 permutations, and pairwise tests at *p* < 0.05. We conducted Principal Component Analysis (PCA) to reduce dimensionality in the fine-scale riverine landscape patch dataset (i.e., environmental patch data) and retained principal components with eigenvalues >1 as predictors of ant density and diversity in subsequent mixed stepwise linear regression models [53,54]. Lastly, we used Moran’s *I* to test for potential spatial autocorrelation among ant assemblage descriptors, whereby *p* > 0.05 indicates a random spatial distribution. PCA and regression analyses were run in JMP 11.0 (SAS Institute Inc., Cary, North Carolina), Moran’s *I* was run in ArcGIS 10.1, and the remaining analyses were conducted in R Software [55], with NMDS, ANOSIM, and SIMPER run using the “vegan” package.

## Results

In total, we delineated 253 riverine landscape patches across all study reaches. The distribution of patches was uneven across the 12 reaches and the three LULC classes, with woody vegetation patches numerically dominant across LULC classes (Table 3). Shrub and swamp patches also occurred in all three LULC classes but collectively constituted a small percentage of the 253 patches (Table 3). We identified lawn patches only in developed reaches, although they represented a small percentage of the total number of patches in developed riverine landscapes. Mixed riverine landscapes were the largest by area, constituting 1,132.8 ha, followed by agricultural (729.1 ha) and developed (503.0 ha) reaches. By patch type, woody vegetation represented the largest area, representing 63% of collective riverine landscape area. In contrast, gravel patches represented only ~1% of land area across the riverine landscapes.

**Table 3:** Summary statistics of ants surveyed by riverine landscape land-use class (agriculture, mixed, developed) including total ant abundance and mean and standard deviation of density and diversity measures by patch type.

*Land Use*	Patch Type and Number	Ant Abun-dance	Ant Density (m^-2^)	Ant Richness (*S*)	Menhinick’s Index (*D*_*M*_)	Berger-Parker Index (*D*_*BP*_)
*Agriculture (n = 2)*		*406*	*1*.*65 ± 2*.*88*	*0*.*61 ± 0*.*66*	*0*.*46 ± 0*.*38*	*0*.*45 ± 0*.*47*
	Crop (15)	141	3.13 ± 5.19	0.73 ± 0.70	0.39 ± 0.30	0.57 ± 0.48
	Grass (5)	26	1.73 ± 2.14	0.80 ± 0.84	0.57 ± 0.37	0.59 ± 0.54
	Gravel (2)	0	0.00 ± 0.00	0.00 ± 0.00	0.00 ± 0.00	1.00 ± 0.00
	Shrub (8)	23	0.96 ± 1.43	0.38 ± 0.52	0.14 ± 0.20	0.38 ± 0.52
	Swamp (2)	10	1.67 ± 2.36	1.00 ± 1.41	0.95 ± 0.07	0.45 ± 0.64
	Woody veg. (50)	206	1.37 ± 2.09	0.60 ± 0.64	0.53 ± 0.39	0.43 ± 0.45
*Mixed* *(n = 5)*		*4*,*466*	*7*.*23 ± 9*.*98*	*1*.*41 ± 1*.*06*	*0*.*54 ± 0*.*35*	*0*.*66 ± 0*.*42*
	Crop (36)	663	6.13 ± 6.50	1.28 ± 1.34	0.49 ± 0.31	0.57 ± 0.45
	Grass (11)	238	7.21 ± 6.34	1.64 ± 1.03	0.49 ± 0.23	0.68 ± 0.42
	Gravel (17)	4	7.92 ± 6.39	1.89 ± 1.11	0.60 ± 0.27	0.76 ± 0.35
	Mudflat (1)	65	21.67 ± —	1.00 ± —	0.25 ± —	0.98 ± —
	Shrub (4)	62	5.16 ± 6.45	1.25 ± 1.50	0.30 ± 0.37	0.49 ± 0.56
	Swamp (8)	169	7.04 ± 4.13	1.75 ± 0.46	0.57 ± 0.39	0.93 ± 0.10
	Woody veg. (126)	3,265	7.41 ±11.69	1.36 ± 1.04	0.56 ± 0.38	0.66 ± 0.42
*Developed (n = 5)*		*3*,*406*	*6*.*55 ±11*.*67*	*1*.*37 ± 1*.*01*	*0*.*54 ± 0*.*32*	*0*.*65 ± 0*.*40*
	Gravel (2)	5	10.83 ±0.24	2.00 ± 0.00	0.60 ± 0.11	0.94 ± 0.04
	Lawn (26)	641	8.22 ±17.30	1.16 ± 1.01	0.46 ± 0.32	0.58 ± 0.45
	Mudflat (19)	220	3.66 ± 2.94	1.50 ± 1.10	0.61 ± 0.30	0.67 ± 0.43
	Shrub (6)	149	8.28 ± 7.84	1.33 ± 0.82	0.46 ± 0.37	0.59 ± 0.47
	Swamp (25)	389	5.18 ± 5.41	1.56 ± 1.00	0.56 ± 0.28	0.74 ± 0.36
	Woody veg. (96)	2,002	6.86 ±12.45	1.35 ± 1.02	0.54 ± 0.33	0.65 ± 0.39
*All reaches*		*8*,*278*	*5*.*98 ±10*.*05*	*1*.*25 ± 1*.*02*	*0*.*53 ± 0*.*35*	*0*.*62 ± 0*.*43*

Note that not all patch types were observed in all three riverine land-use classes.

Patch metrics were highly variable both within and across LULC classes (Table 4). Total Landscape Area (TLA, including the main channel area) was 166% greater in agricultural than in developed reaches (ANOVA: *F* = 6.23, *p* = 0.020; Tukey HSD: *p* = 0.016). A number of other notable, although non-significant relationships emerged including that Mean Patch Size (MPS)—another metric describing patch area—was 46% greater in agricultural than in developed reaches and that patch density ([represented by Number of Patches [NP]) was ~140% greater in developed than in either mixed or agricultural riverine landscapes (Table 4).

**Table 4:** Summary statistics of patch metrics for all twelve study riverine landscapes as well as summary statistics for patches broken out by the three land-use classes.

Patch Metric	Overall		Agriculture		Mixed		Developed	
	Mean	SD	Mean	SD	Mean	SD	Mean	SD
*Area metrics*								
Total Landscape Area (TLA)	47.02	25.31	85.54	18.67	46.57	12.96	32.05	21.98
Mean Patch Size (MPS)	31.87	12.70	37.29	17.14	36.12	6.77	25.42	15.31
*Density metrics*								
Number of Patches (NP)	14.50	6.20	12.50	7.80	12.40	6.20	17.40	5.90
*Edge metrics*								
Edge Density (ED)	157.09	63.08	129.70	50.20	128.10	24.20	197.00	79.40
Mean Patch Edge (MPE)	5.30	1.36	5.28	0.42	5.61	1.81	5.01	1.26
Total Edge (TE)	73.62	31.53	67.60	46.29	62.09	9.76	87.55	0.41
Mean Perimeter Area Ratio (MPAR)	345.40	164.90	279.30	20.30	243.30	52.90	473.90	189.80
*Diversity metrics*								
Shannon Diversity Index (SDI)	1.39	0.28	1.21	0.47	1.44	0.20	1.43	0.32
Shannon Evenness Index (SEI)	0.76	0.08	0.69	0.08	0.80	0.06	0.75	0.10
*Shape metrics*								
Mean Shape Index (MSI)	2.90	0.71	2.43	0.00	2.76	0.87	3.20	0.63

Note that values for MPE and TE were scaled down by a factor of 1,000.

### Effects of LULC vs. riverine landscape patches on ant assemblage density and diversity

We surveyed 8,278 ants at 459 quadrats representing 10 genera and 23 species (S1 Table). A species accumulation curve for the study system plateaued by 215 sampling points (i.e., quadrats, out of 459 total), giving us confidence that our sampling effort was adequate (S1 Fig). The most numerically dominant species (from greatest to least) were: *T*. *sessile* (3,393 individuals), *A*. *tennesseensis* (2,024 individuals), and *F*. *subsericea* (1,925 individuals), which collectively represented 89% of the ant community. We observed marked variability in ant abundance, density, and diversity measures both within and among riverine landscapes (Table 3). Fifty-four percent of all ants (i.e., abundance) was found in mixed riverine landscapes, followed by 41% in developed, and 5% in agricultural reaches (Table 3). We found no evidence for spatial autocorrelation in patterns of ant assemblages (Moran’s *I*; *p* > 0.05).

The results of nested ANOVAs indicated that there was significant variation in ant assemblage density (*F* = 7.05, *p* = 0.018), *S* (*F* = 12.48, *p* < 0.002), and the Berger-Parker Index (*D*_*BP*_: *F* = 5.97, *p* = 0.021) among LULC classes, but not among riverine landscape patches within study reaches (*p* > 0.05). Mean ant density was >300% times lower at agricultural (1.65 ± 2.08 ind. m^2^) than at mixed (7.23 ± 9.98 ind. m^2^; Tukey HSD: *p* = 0.011) and developed (6.55 ± 11.67 ind. m^2^; Tukey HSD: *p* = 0.080) riverine landscapes. In contrast, *D*_*BP*_ was ~145% lower in agricultural (0.45 ± 0.47) than in both developed (0.65 ± 0.40; Tukey HSD: *p* = 0.008) and mixed (0.66 ± 0.42; Tukey HSD: *p* = 0.002) riverine landscapes but not significantly different among patch types (*p* > 0.05) (note that lower *D*_*BP*_ values indicate higher diversity). *S* was lower in agricultural (0.61 ± 0.66) versus developed (1.37 ± 1.01; Tukey HSD: *p* = 0.0002) and mixed (1.41 ± 1.06; Tukey HSD: *p* < 0.0001) riverine landscapes, respectively, but not different among patch types (*p* > 0.05). Menhinick’s Index was not significantly different among LULC or patch types (*p* > 0.05).

Although no significant differences in ant density or diversity measures were found among riverine landscape patches, evidence suggested that patch metrics (i.e., edge, shape, etc.) influenced multiple ant assemblage descriptors. PCA identified four axes (eigenvalues >1) that accounted for ~97% of the variation in the patch-metric dataset (Table 5). We named each of the four axes after those patch metrics that predominantly loaded on the PCs, hence we had “Area/Edge Axis” for PC1, “Density Axis” for PC2, “Shape Axis” for PC3, and “Diversity Axis” for PC4 (Table 5). Using these PCA axes as predictor variables for ant assemblages at the patch scale yielded significant models for univariate ant metrics. Ant density was significantly predicted by a combination of the Area/Edge and the Density Axes to explain 65% of the variation (*F* = 7.46, *p* = 0.012). Area/Edge and Shape Axes together accounted for 62% of the variation observed in *D*_*BP*_ (*F* = 7.33, *p* = 0.013). Shape and Density axes jointly predicted ant species richness assessed using both *S* (*R* ^*2*^ = 0.72, *F* = 11.58, *p* = 0.003) and *D*_*M*_ (*R* ^*2*^ = 0.57, *F* = 6.00, *p* = 0.022).

**Table 5:** Eigenvalues (>1.0) and the percent variance captured by the principal components (PCs) along with the loadings.

Patch Metric	PC1	PC2	PC3	PC4
	Area/Edge Index	Density Index	Shape Index	Diversity Index
Edge Density	**-0.41**	0.19	0.30	0.07
Mean Patch Size	**0.42**	-0.30	0.08	0.16
Mean Perimeter Area Ratio	**-0.37**	0.24	0.31	0.12
Total Land Area	**0.45**	0.17	0.08	0.26
Number of Patches	0.18	**0.57**	0.06	0.12
Mean Patch Edge	0.15	**-0.45**	0.37	0.31
Total Edge	0.32	0.33	0.30	0.32
Mean Shape Index	-0.27	-0.15	**0.54**	0.20
Shannon Diversity Index	0.23	0.30	0.31	**-0.57**
Shannon Evenness Index	0.18	-0.22	0.03	**-0.56**
Eigenvalue	4.07	2.54	2.03	1.06
*% variance*	40.66	25.38	20.34	10.60

Bold print represents the most influential loadings for each eigenvector. Names assigned to each PC axis represent these influential loadings.

### Associations between patch type and ant assemblage composition

**Fig 3:**
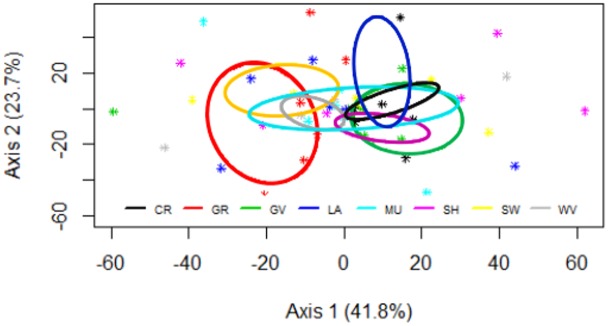
Non-metric multidimensional scaling (NMS). NMS plots showing dissimilarity matrices of the collective relative abundance of all ant species surveyed (stress value = 0.18). Points represent class centroids (i.e., weighted means) of ant relative abundance in each patch type of each study reach (*n* = 49). The amount of variation represented by each axis is indicated in parentheses. The ellipses indicate 95% confidence intervals for clusters of each patch type and show separation in ant assemblage composition in grass/herbaceous and swamp from crop, gravel, lawn and shrub as well as woody vegetation from crop, lawn, and gravel. CR = crop, GR = grass/herbaceous, GV = gravel, LA = lawn, MU = mudflat, SH = shrub, SW = swamp, and WV = woody vegetation.

NMS ordination distinguished ant assemblage composition in **gravel and swamp patches from assemblages in crop, gravel, lawn, and shrub along the first axis, and assemblages in woody vegetation patches from those in crop, lawn, and gravel** along the second axis (stress = 0.18, *R* ^2^ = 0.64; Fig 3). ANOSIM supported these results, revealing significant (*r* = 0.63, *p* = 0.030) differences in ant assemblage composition at the patch level. *A*. *tennesseensis*, *F*. *subsericea*, and *T*. *sessile* contributed greatest to dissimilarity between land-use pairs, cumulatively accounting for 72.1% between developed and agricultural, 77.6% between developed and mixed, and 77.7% between mixed and agricultural land uses (S2 Table). However, NMS did not distinguish ant assemblage composition among the three LULC types (stress = 0.31, *p* > 0.05; S2 Fig).

## Discussion

In spite of high variability in internal riverine landscape patch heterogeneity, broader landscape characteristics related to LULC appeared to drive ant assemblage abundance and diversity in our study system. Although ant density and diversity were largely invariant among riverine landscape patches, we observed shifts in ant assemblage composition among patches, with assemblage composition in grass/herbaceous, swamp, and woody vegetation patches the most distinct. Collectively, our study provides evidence that both landscape- and local-habitat characteristics contribute to explaining patterns in riparian ant assemblages in the Scioto basin and represents a valuable application of landscape ecology to ecological communities in river corridors.

### Effects of LULC vs. riverine landscape patches on ant assemblage density and diversity

Variability in LULC relationships with ant density and diversity observed in our study may point to the geographic reduction of some species (losers) and the expansion of others (winners) as landscapes are transformed from rural to urban [56]. In our study, for example, although ant species richness (*S*, but not *D*_*M*_) was greater at developed and mixed riverine landscapes than agricultural reaches, Berger-Parker Index was higher at these reaches, indicating lower community diversity and potential simiplification of the ant assemblage via dominance of a few species—results generally consistent with Thompson and McLachlan [46]. The significantly higher ant assemblage density we observed in both developed and mixed reaches as compared to agricultural reaches also align with results of past studies. For example, Lessard and Buddle [57] observed higher ant abundance in urban areas than in protected forests in Quebec, Canada.

However, other findings contrast our results. Ives, Taylor, Nipperess and Hose [58] observed no significant difference in riparian ant diversity and assemblage structure between urban and rural catchments in Sydney, Australia. Additionally, Buczkowski and Richmond [59] report the loss of 17 ant species after urban construction in West Lafayette, Indiana, USA. *T*. *sessile* was the second most frequently encountered ant species in Buczkowski and Richmond’s [59] Indiana study and the most commonly encountered species in our Ohio study, where it also was the most influential species relative to assemblage dissimilarity between all land-use pairings (developed-mixed: 37.2%, developed-agriculture: 34.7%, mixed-agriculture: 38.5%; S2 Table). Neither the dominance nor the influence on community turnover among land-use types is surprising given that *T*. *sessile* has the greatest ecological tolerance of any ant in North America, is commonly found in both natural and man-made habitats [48], and can exhibit invasive characteristics in developed settings [60].

Associations between ant diversity and density are likely related to multiple mechanisms operating at both the landscape and patch scales. In some cases, for example, invertebrate species abundance has been shown to increase with structural complexity of the environment [61,62]. As such, disturbances such as periodic inundation in riverine landscapes often produce patches with dissimilar habitat characteristics (e.g., soil moisture and soil temperature [63,64]) which can lead to filtering of riverine arthropod abundance and composition assemblage [26]. Secondly, although our study did not directly investigate temperature, the concept of urban heat gradients is well established [e.g., 65], and may implicate temperature as a driver of high ant density in urban reaches of our study system. Specifically, riparian environments in developed and mixed landscapes may be more attractive to ants in part because of greater light availability and relatively high soil temperature [66]. Because ants are generally thermophilic [67], their abundance often increases with increasing temperature [68], which could partly explain why some ant species—including *F*. *subsericea* and *T*. *sessile*, together representing 41% of the urban ant fauna in our study—tend to be closely associated with human activities [69]. These species can affect local community ant diversity through competitive or exploitative interactions [70], which also might be a factor contributing to the lower Berger-Parker Index (i.e., higher diversity and greater assemblage evenness) values we observed in agricultural than in either developed or mixed riverine landscapes. Lastly, the lower density of ants in riverine landscapes embedded in agricultural landscapes is consistent with the observation by Petal [71] that fertilization of farmland can lead to a reduction in ant density as mineral fertilizers and chemicals that may be toxic to ants are commonly used in agricultural practices in the Scioto River basin [72].

Although we found greater evidence for the influence of LULC than internal riverine landscape patch type on ant density and diversity measures, patch metrics quantifying patch area, edge, shape, and density resulted in models that explained >50% of the variation in ant density and diversity. Pluralistic explanations for the relationships between patch configuration and ant assemblage characteristics are likely. Patch edges can alter the flows of energy and organisms [73] and lead to changes in ant density gradients near and along edges [74]. Edges also often have dissimilar soil moisture and soil temperature regimes from those of core areas [73,75]. Thus, patch geometry and amount of edge might be expected to be important in structuring the distribution of arthropods [76,77]. Patch density (a proxy for habitat diversity) may influence ant density and diversity via the mechanisms suggested by the habitat heterogeneity hypotheses [e.g., 78], whereby high habitat heterogeneity leads to higher diversity of species. Because larger area usually facilitates greater diversity of organisms [79,80], it is not surprising that larger patches were associated with higher ant diversity than smaller patches in our study system.

### Effects of patch type on ant assemblage composition

The partitioning of ant assemblage composition (i.e., relative abundance) by specific patch types supported our hypotheses and pointed to the importance of riverine landscape patch heterogeneity to ant assemblage diversity. Ant assemblages in woody vegetation patches, for example, were distinct from those in crop, lawn, and gravel, supporting the role that forests provide important habitat for many arthropods including ants [81]. The most dominant ant species we sampled *(T*. *sessile*, *A*. *tennesseensis*, *F*. *subsericea)* prefer to nest in vegetated habitats [31,82] with snags and tree cavities [81], which were more ubiquitous in woody vegetation patches than the other patch types. Moreover, the frequent and stochastic flooding typical of mudflats, swamps, and gravel bars can limit ant abundance and diversity [83]. Microclimatic conditions of gravel bars—particularly pertaining to temperature extremes—may also be limiting to many species of ants, whereas agricultural chemicals and tillage activities may favor more tolerant species over others and lead to shifts in relative abundance within the ant community in cropland patches [71].

In this study, we used relative measures of abundance and diversity of a subset of the ant community (diurnal, surface active) to investigate riverine landscape patch dynamics. Nonetheless, although our sampling effort was adequate for our objectives, increasing the sampling effort through inclusion of other survey methods (e.g., pitfall traps, sticky traps and baits, nocturnal surveys) may yield further insight into the effects of riparian patchiness on arthropod biodiversity. In particular, we may have missed more secretive members of the ant community (e.g., *Brachmyrmex depilis*, *Stigmatomma pallipes*, and a few species of hypogaeic *Lasiu*s, *Ponera*, and *Hypoponera*), which are likely present at these sites (Kal Ivanov, personal communication). Given both natural (e.g., hydrologic) and human-induced (e.g., urbanization, agriculture, etc.) disturbance characteristics of our riparian study system, invasive, exotic species might also have been expected to play an important role [e.g., 84]. *Tetramorium caespitum* (European species), for example, is widespread in Ohio and highly abundant in human-disturbed areas [85]. However, *T*. *caespitum* prefers to nest in areas with minimal vegetation [86] and the abundance of *T*. *caespitum* has been shown to be negatively associated with tree density [87], which may partially explain why they were not found in our treed riparian environments. *Nylanderia flavipes* (Asian species), which was first recorded in Ohio in 2005, has been found in riparian corridors of northeastern Ohio, where it can be the numerically dominant species [88], but there is no evidence to date of this species from the southern part of the state. Of note, Ivanov, Lockhart, Keiper and Walton [88] did not observe significant changes in species richness or total abundance of native ants in the presence of *N*. *flavipes*.

## Conclusions

Our results indicate that both broad-scale landscape features as well as finer-scale patch dynamics contribute to explaining variation in the density, diversity, and composition of riparian ant assemblages. We recognize that other variables (e.g., soil moisture and soil temperature, cross-boundary food subsidies) may also be important in governing ant assemblage characteristics. As such, future studies should assess variables including microclimatic conditions and food-resource availability and analyze these against ant assemblage characteristics. Explicit investigation of the mechanisms linking landscape and local habitat-arthropod associations will also be an important direction for future research. Nevertheless, our research advances current understanding of the utility of landscape ecology in river-riparian contexts, illustrating that patch context (i.e., LULC class) and patch quality (e.g., size, shape, edge characteristics) have important ecological implications. For example, because ants may also be agents in the propagation of plants via seed dispersal [45], the influence of patchiness on ant distribution may influence ant-mediated plant seed dispersal. Our findings represent an important step in integrating river science with landscape ecology and provide insight into riverine landscape conservation in managed landscapes.

## Supporting Information

S1 Fig Species accumulation curves. The jagged lines are the species accumulation curves for 459 ant sampling quadrats, yielding a total of 8,278 individual ants and 23 species from an intensive survey of surface-active ants of 12, 1,200-m riverine landscapes grouped by land-use and land-cover types (developed, mixed, and agriculture) along the Scioto River, Ohio, USA. The cumulative number of ant species (y axis) is plotted as a function of the cumulative number of samples (x axis), pooled in random order.
(TIFF)

S2 Fig Non-metric multidimensional scaling (NMS). NMS plots showing dissimilarity matrices of the collective relative abundance of the three most abundant ant species (stress value = 0.31, *p* > 0.05). Points represent class centroids (i.e., weighted means) of ant relative abundance in each patch type of each study reach (*n* = 49). The amount of variation represented by Axis 1 is 32.3% and by Axis 2 is 21.3%. The ellipses indicate 95% confidence intervals for clusters of each patch type and show no separation in ant assemblage composition among LULC types.
(TIFF)

S1 Table Species distributions. Species distributions (relative abundance) by land cover and patch type, along with lat/longs for each quadrat/sample location.
(XLSX)

S2 Table Similarity percentage (SIMPER) analysis. Similarity percentage (SIMPER) analysis representing the average % contribution of each species to the dissimilarity (individual [cont %] and cumulative total [cum %]) in species abundance between each pair of land-use types.
(DOC)
