# Supplementary figures and images for: Riverine Landscape Patch Heterogeneity Drives Riparian Ant Assemblages in the Scioto River Basin, USA
Source: PLoS One. 2015 Apr 20;10(4):e0124807. doi: 10.1371/journal.pone.0124807 (PMC4403917; doi:10.1371/journal.pone.0124807)

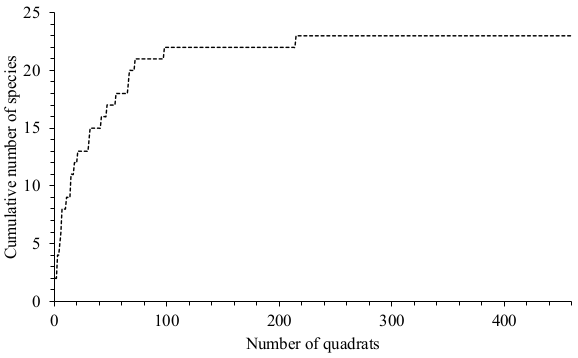

Supplement: S1 Fig — The jagged lines are the species accumulation curves for 459 ant sampling quadrats, yielding a total of 8,278 individual ants and 23 species from an intensive survey of surface-active ants of 12, 1,200-m riverine landscapes grouped by land-use and land-cover types (developed, mixed, and agriculture) along the Scioto River, Ohio, USA. The cumulative number of ant species (y axis) is plotted as a function of the cumulative number of samples (x axis), pooled in random order. (TIFF) [file pone.0124807.s001.tiff]

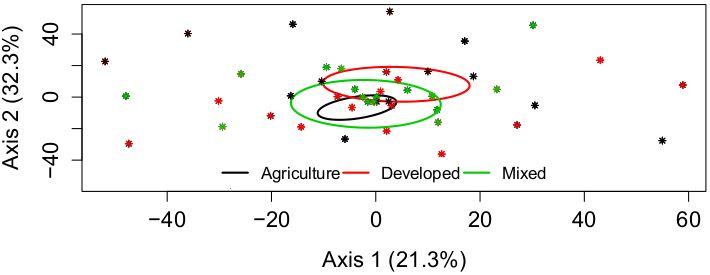

Supplement: S2 Fig — NMS plots showing dissimilarity matrices of the collective relative abundance of the three most abundant ant species (stress value = 0.31, p > 0.05). Points represent class centroids (i.e., weighted means) of ant relative abundance in each patch type of each study reach (n = 49). The amount of variation represented by Axis 1 is 32.3% and by Axis 2 is 21.3%. The ellipses indicate 95% confidence intervals for clusters of each patch type and show no separation in ant assemblage composition among LULC types. (TIFF) [file pone.0124807.s002.tiff]
