# Supplementary material for: Riverine Landscape Patch Heterogeneity Drives Riparian Ant Assemblages in the Scioto River Basin, USA
Source: PLoS One. 2015 Apr 20;10(4):e0124807. doi: 10.1371/journal.pone.0124807 (PMC4403917; doi:10.1371/journal.pone.0124807)
Supplement: S2 Table — Similarity percentage (SIMPER) analysis representing the average % contribution of each species to the dissimilarity (individual [cont %] and cumulative total [cum %]) in species abundance between each pair of land-use types. (DOC) [file pone.0124807.s004.doc]

| **Species** | **Developed-Mixed** | |  | **Developed-Agriculture** | |  | **Mixed-Agriculture** | |
| --- | --- | --- | --- | --- | --- | --- | --- | --- |
|  | Cont (%) | Cum (%) |  | Cont (%) | Cum (%) |  | Cont (%) | Cum (%) |
| *Tapinoma sessile* | 37.23 | 37.23 |  | 34.73 | 34.73 |  | 38.52 | 38.52 |
| *Formica subsericea* | 24.42 | 61.65 |  | 22.79 | 57.52 |  | 28.64 | 67.16 |
| *Aphaenogaster tennesseensis* | 15.90 | 77.55 |  | 14.55 | 72.07 |  | 10.52 | 77.68 |
| *Formica exsectoides* | 6.13 | 83.68 |  | 5.59 | 77.66 |  | 2.57 | 80.25 |
| *Solenopsis molesta* | 1.42 | 85.10 |  | 2.32 | 79.98 |  | 2.5 | 82.75 |
| *Myrmica punctiventris* | 1.42 | 86.52 |  | 2.27 | 82.25 |  | 2.17 | 84.92 |
| *Formica sp.* | 1.40 | 87.92 |  | 2.21 | 84.46 |  | 1.57 | 86.49 |
| *Temnothorax curvispinosus* | 1.40 | 89.32 |  | 2.08 | 86.54 |  | 1.56 | 88.05 |
| *Camponotus subbarbatus* | 1.30 | 90.62 |  | 1.87 | 88.41 |  | 1.55 | 89.60 |
| *Crematogaster cerasi* | 1.20 | 91.82 |  | 1.77 | 90.18 |  | 1.55 | 91.15 |
| *Aphaenogaster fulva* | 1.18 | 93.00 |  | 1.36 | 91.54 |  | 1.53 | 92.68 |
| *Camponotus pennsylvanicus* | 0.95 | 93.95 |  | 1.31 | 92.85 |  | 1.36 | 94.04 |
| *Proceratium silaceum* | 0.87 | 94.82 |  | 1.22 | 94.07 |  | 1.05 | 95.09 |
| *Camponotus nearcticus* | 0.82 | 95.64 |  | 1.04 | 95.11 |  | 0.91 | 96.00 |
| *Aphaenogaster treatae* | 0.75 | 96.39 |  | 0.92 | 96.03 |  | 0.92 | 96.92 |
| *Temnothorax longispinosus* | 0.74 | 97.13 |  | 0.86 | 96.89 |  | 0.77 | 97.69 |
| *Camponotus novaeboracensis* | 0.68 | 97.81 |  | 0.81 | 97.70 |  | 0.76 | 98.45 |
| *Myrmica americana* | 0.64 | 98.45 |  | 0.80 | 98.50 |  | 0.71 | 99.16 |
| *Solenopsis texana* | 0.61 | 99.06 |  | 0.54 | 99.04 |  | 0.61 | 99.77 |
| *Camponotus sp.* | 0.37 | 99.43 |  | 0.49 | 99.53 |  | 0.15 | 99.92 |
| *Lasius neoniger* | 0.25 | 99.68 |  | 0.31 | 99.84 |  | 0.06 | 99.98 |
| *Formica sp.* | 0.19 | 99.87 |  | 0.11 | 99.95 |  | 0.01 | 99.99 |
| *Crematogaster pilosa* | 0.13 | 100.00 |  | 0.01 | 100.00 |  | 0.01 | 100.00 |
